# Simultaneous Oxidation of Atmospheric Methane, Carbon Monoxide and Hydrogen for Bacterial Growth

**DOI:** 10.3390/microorganisms9010153

**Published:** 2021-01-12

**Authors:** Alexander Tøsdal Tveit, Tilman Schmider, Anne Grethe Hestnes, Matteus Lindgren, Alena Didriksen, Mette Marianne Svenning

**Affiliations:** 1 Department of Arctic and Marine Biology, UiT, The Arctic University of Norway, 9037 Tromsø, Norway; tilman.schmider@uit.no (T.S.); anne.hestnes@uit.no (A.G.H.); alena.didriksen@uit.no (A.D.); mette.svenning@uit.no (M.M.S.); 2 CAGE—Centre for Arctic Gas Hydrate, Environment and Climate, Department of Geosciences, UiT, The Arctic University of Norway, 9010 Tromsø, Norway; matteus.lindgren@uit.no

**Keywords:** methane, carbon monoxide, hydrogen, energy, growth, atmospheric trace gases

## Abstract

The second largest sink for atmospheric methane (CH_4_) is atmospheric methane oxidizing-bacteria (atmMOB). How atmMOB are able to sustain life on the low CH_4_ concentrations in air is unknown. Here, we show that during growth, with air as its only source for energy and carbon, the recently isolated atmospheric methane-oxidizer *Methylocapsa gorgona* MG08 (USCα) oxidizes three atmospheric energy sources: CH_4_, carbon monoxide (CO), and hydrogen (H_2_) to support growth. The cell-specific CH_4_ oxidation rate of *M. gorgona* MG08 was estimated at ~0.7 × 10^−18^ mol cell^−1^ h^−1^, which, together with the oxidation of CO and H_2_, supplies 0.38 kJ Cmol^−1^ h^−1^ during growth in air. This is seven times lower than previously assumed necessary to support bacterial maintenance. We conclude that atmospheric methane-oxidation is supported by a metabolic flexibility that enables the simultaneous harvest of CH_4_, H_2_ and CO from air, but the key characteristic of atmospheric CH_4_ oxidizing bacteria might be very low energy requirements.

## 1. Introduction

Atmospheric methane-oxidizing bacteria (atmMOB) remove 9–47 Tg methane (CH_4_) from the atmosphere annually [1]. The most common hypothesis for explaining how these bacteria can sustain life by oxidizing atmospheric CH_4_ is the “high affinity” model, in which a special form of particulate CH_4_ monooxygenase allows atmMOB to oxidize the low atmospheric CH_4_ concentrations at a high rate [2]. Other hypotheses include the utilization of alternative energy sources or a high specific affinity (V_max(app)_/*K_m(app)_*) [3,4]. AtmMOB are found globally in soils [4] and belong to the two phylogenetic clusters USCα (Alphaproteobacteria) and USCγ (Gammaproteobacteria) [5]. The lack of pure cultures from USCα and USCγ has prevented studies of the energy metabolism of atmMOB [3]. Conventional methane oxidizing bacteria (MOB) are expected to oxidize around 0.2–17 × 10^−18^ mol CH_4_ cell^−1^ h^−1^ at atmospheric CH_4_ concentrations at 25 °C, but such rates have been considered too low to provide sufficient energy for growth [6]. Thus, atmMOB were expected to either utilize alternative energy sources or have high affinities for CH_4_ [3]. The latter is supported by observations reporting apparent high affinity CH_4_ oxidation by microbial communities in oxic soils [7]. Additionally, oxidation rates of 800 × 10^−18^ mol CH_4_ cell^−1^ h^−1^ were measured in forest soils, far surpassing the cell-specific activity of 40 × 10^−18^ mol CH_4_ cell^−1^ h^−1^ assumed to be necessary to support minimum cell maintenance requirements [6,8]. At 20 °C, these minimum requirements would demand an energy supply of 2.8 kJ Cmol h^−1^. It is assumed that the high forest soil rates at atmospheric CH_4_ concentrations are achieved by the combination of methane monooxygenases (MMO) with very low half-saturation constants (K_m_) and high cellular abundances of these enzymes.

The recently described USCα atmMOB species *Methylocapsa gorgona* MG08 has the highest specific affinity (*V*_max(app)_/*K_m(app)_*) for CH_4_ of all tested MOB at 195 × 10^−12^ L⋅cell^−1^⋅h^−1^. However, this translates into a cell-specific CH_4_ oxidation rate of only 10 × 10^−18^ mol CH_4_ cell^−1^ h^−1^ at atmospheric CH_4_ concentration [4], seemingly too low to support cellular maintenance [6]. Nevertheless, *M. gorgona* MG08 is able to grow at atmospheric (1.87 p.p.m.v.) CH_4_ concentrations [4]. It also carries a [NiFe] group 1 h high-affinity respiratory hydrogenase (*hhyL* and *hhyS*) and a [MoCu] class I respiratory carbon monoxide dehydrogenase, similar to those identified in atmospheric carbon monoxide (CO) and hydrogen (H_2_)-oxidizing microorganisms [4,9,10,11,12]. Recent studies have identified the utilization of atmospheric CO and H_2_ as energy sources for growth and survival in bacteria [9,11,12,13], and as support for bacterial primary production in Antarctic and Arctic environments [10,14]. Atmospheric CH_4_ concentrations are currently 1.87 p.p.m.v. [15], while the concentrations of atmospheric CO and H_2_ are lower. CO concentrations vary, with estimates for uninhabited areas around 0.1 p.p.m.v., while urban areas contain higher concentrations, often above 0.2 p.p.m.v. [16,17,18,19]. Atmospheric H_2_ concentrations are stable at approximately 0.53 p.p.m.v. [20]. Many MOB carry genes for carbon monoxide and hydrogen oxidation [4], and recently it has been shown that the thermoacidophilic *Methylacidiphilum fumariolicum* SolV can oxidize sub-atmospheric H_2_ with a high-affinity, membrane-associated [NiFe] hydrogenase [21], while the strain *Methylocystis* sp. SC2 oxidizes hydrogen at higher concentrations [22]. However, the oxidation of atmospheric CO and H_2_ to support growth has never been demonstrated for *M. gorgona* MG08 or any other MOB. We have studied how the atmospheric CH_4_ oxidizer *M. gorgona* MG08, in pure culture, harvests energy from the atmosphere for growth.

## 2. Materials and Methods

### 2.1. Cultivation

A young stationary culture of *M. gorgona* MG08 was prepared as follows. A 20 mL culture (1:10 (NMS:MilliQ) in diluted liquid nitrate mineral salts (NMS) medium (pH 6.8) (DSMZ medium 921) without EDTA) with a headspace of 20% CH_4_ in air (20 mL 100% CH4 mixed with 80 mL of air) was incubated for 12 days, reaching several days (2–4) into the stationary phase. Inoculum from this culture was precultured in 1:10 (NMS:MilliQ) diluted liquid nitrate mineral salts (NMS) medium (pH 6.8) (DSMZ medium 921) without EDTA under a headspace atmosphere of ~20 p.p.m.v. CH_4_ in air for two weeks. The headspace was created by injecting 20 µL of 100% CH_4_ into the 100 mL ambient air headspace of 120 mL serum bottles containing 20 mL medium, sealed with a butyl rubber stopper and crimp cap. This was used as a start culture for colony growth on filters. The cell concentration in the suspension was determined by fluorescence microscopy after filtration on Anodisc filters (Whatman 6809-6022, Merck, Darmstadt, Germany) and 1000× SYBRgreen I (Molecular probes S-7567, ThermoFisher, Waltham, MA, USA) staining as previously described [4]. For staining, filters were transferred (bacteria side up) on top of 200 µL droplets of 1000× SYBRgreen and incubated for 10 minutes. In the next step, filters were washed twice by transferring them onto 1 mL milliQ water and then air-dried. The whole procedure was performed at room temperature and in the dark. A fresh-made anti-fading solution consisting of 0.1% p-phenylenediamine dihydrochloride in 1:1 glycerol and PBS (phosphate buffered saline pH 7.2) was used for mounting the filters on slides with cover slips.

From the start cultures, the required volume and cell density needed to achieve a cell density of ~20 cells per 63× photo area, for a total of ~1 × 10^6^ cells per filter, was selected for filter cultivation. For the transfer of cells onto the 47 mm polycarbonate filter, an autoclaved Millipore vacuum filter holder system (cat no XX1004700, Merck, Darmstadt, Germany) with 300 mL glass funnels was used. Firstly, a GF/C filter (Whatman 1822-047, Merck, Darmstadt, Germany) was added as support filter. A polycarbonate filter (Whatman, Nucleopore 111106, Merck, Darmstadt, Germany) was then added and the funnel was clamped on top. Even distributions of cells on the filters were obtained by pouring 100 mL of autoclaved milliQ-water into the funnel before mixing in 0.1 mL of the start culture cell suspension and applying vacuum. The funnel walls were rinsed twice with 15 mL sterile water during suction. The polycarbonate filters were then transferred into 250 mL bottles (DURAN, Merck, Darmstadt, Germany) in a Holten LaminAir bench and left floating on 50 mL of 1:10 (NMS:MilliQ) diluted liquid NMS medium (pH 6.8) without EDTA. Finally, the bottles were covered with gas permeable parafilm (Merck, Darmstadt, Germany). These bottles were incubated in a ventilated room at 20 °C in darkness for nine months (experiment 1) or 4.5 months (experiment 2), after which the gas uptake experiments were initiated. In Experiment 1, an additional inspection of a filter was performed after eight months. This included a visual inspection and counting of 60 colonies on two different filters to show that the number of generations in almost all growing colonies had surpassed seven (64 cells), indicating that the filters had sufficient biomass to initiate gas uptake experiments.

### 2.2. Temperature Selection

All cultivations and experiments were carried out at 20 °C. This temperature was chosen as it is in the middle of the optimum temperature range for growth (~15–~27 °C) of *M. gorgona* MG08 and was the temperature at which previous experiments were carried out [4].

### 2.3. Gas Uptake and Leakage Experiments

Three experiments were carried out: two gas uptake experiments that included controls and one control experiment. Experiment 1 consisted of 53 × 120 mL bottles, some containing cultures of *M. gorgona* MG08 on filters floating on liquid media. Experiment 2 consisted of 15 bottles. The control experiment consisted of 18 bottles.

In all experiments, the bottles contained 50 mL of 1/10 diluted NMS medium without EDTA. All gases used were of the highest available quality (6.0) (all gases were supplied by AGA). For the first gas uptake experiment, a total of 7 different setups (A–G) of five replicates each were created: A–E contained filters with *M. gorgona* MG08 cells, and F and G did not contain *M. gorgona* MG08 cells. (A) five bottles with 2–4 p.p.m.v. CH_4_, 2–4 p.p.m.v. H_2_ and 2–4 p.p.m.v. CO in synthetic air. (B) five bottles with 2–4 p.p.m.v. H_2_ and 2–4 p.p.m.v. CO in synthetic air; (C) five bottles with 2–4 p.p.m.v. CH_4_ in synthetic air. (D) five bottles with an atmosphere of ambient air (compressed outdoor air); (E) five bottles with synthetic air. (F) five bottles with 2–4 p.p.m.v. CH_4_, 2–4 p.p.m.v. H_2_ and 2–4 p.p.m.v. CO in synthetic air. (G) five bottles with ambient air atmosphere.

For the second gas uptake experiment, one condition included cells; five bottles with ~4 p.p.m.v. CH_4_, ~4 p.p.m.v. H_2_ and ~4 p.p.m.v. CO in synthetic air. Two conditions included sterile filters; five bottles with ~4 p.p.m.v. CH_4_, ~4 p.p.m.v. H_2_ and ~4 p.p.m.v. CO in synthetic air and five bottles with synthetic air atmospheres.

The control experiment to determine gas leakages (18 bottles) included six different types of liquids and headspace compositions: (A) 50 mL MilliQ water and 200 mL headspace of pure helium. (B) Sterile floating polycarbonate filter on 50 mL of 1/10 diluted NMS medium without EDTA and 200 mL headspace with 2–5 p.p.m.v. CH_4_, H_2_, CO in synthetic air. (C) 50 mL of milliQ water and synthetic air. (D) 50 mL of 1/10 diluted NMS medium without EDTA and 2–5 p.p.m.v. CH_4_, H_2_, CO in synthetic air. (E) 50 mL of milliQ water and 2–5 p.p.m.v. CH_4_, H_2_, CO in synthetic air. (F) Empty glass bottle with 250 mL of 2–5 p.p.m.v. CH_4_, H_2_, CO in synthetic air.

To create the mentioned atmospheres, the bottles were sealed with halogenated bromobutyl rubber stoppers (DURAN, Merck, Darmstadt, Germany) and plastic screw caps under a sterile bench. Before usage, the rubber stoppers had been boiled ten times and then autoclaved. The sealed bottles were flushed for 15 min with ambient, high quality synthetic air (N_2_: 78%, O_2_: 21%, CO_2_: 400 p.p.m.v.) or pure helium by using a gassing manifold connected to sterile single-use needles. The final pressure in the bottles was adjusted to one bar absolute pressure. Afterwards, 1 mL of additional respective gases (CH_4_, CO and H_2_) was added by using a gas tight syringe (VICI AG International, Schenkon, Switzerland) to create the required atmospheres. To prevent contamination, each gassing step was carried out by interconnecting a sterile 0.2 µm cellulose acetate filter (VWR Collection, Lutterworth, UK) between needle and hose or syringe.

The prepared bottles were incubated at 20 °C for 57 days in the first experiment. The headspace concentrations of CH_4_, CO and H_2_ were measured the day after preparing the headspace atmospheres and after 7, 17 and 57 days. In the second experiment, the incubation lasted 145 h. In the control experiment, the incubations lasted 6, 54 or 70 days.

For combined measurements of CH_4_, CO and H_2_, 1 mL headspace gas was sampled with a gastight GC syringe (VICI AG International, Schenkon, Switzerland). The contained gas was injected manually into a gas chromatograph (ThermoScientific Trace 1310 with column TG-BOND Msieve 5A, ThermoFisher, Waltham, MA, USA). Detection was achieved by using a PDD detector. A high-quality gas containing 5 p.p.m.v. H_2_, 5 p.p.m.v. CH_4_, and 5 p.p.m.v. CO in N_2_ served as standard. To create standard curves, 2 × 0.1 mL, 2 × 0.5 mL, and 2 × 1 mL of the mentioned standard were injected on every measurement day. Bottle gas concentrations were calculated using the standard curve. Masses were calculated by applying the ideal gas law and adjusted for changes in bottle pressure due to gas removal.

### 2.4. Cell Quantification

Due to several layers of cells forming colonies on the filters after 9 months and 57 days of incubation, the number of cells on four filters per condition A–E in the gas uptake experiment could not be estimated reliably by cell counts. Instead, cell numbers were estimated using DNA extractions and comparison to a standard of DNA extraction yields for known cell numbers of *M. gorgona* MG08 on polycarbonate filters. Cells for a standard were prepared as follows. A 20 mL culture (1:10 (NMS:MilliQ) diluted liquid nitrate mineral salts (NMS) medium (pH 6.8) (DSMZ medium 921) without EDTA) with a headspace of 20% CH_4_ in air (20 mL 100% CH_4_ mixed with 80 mL of air) was incubated for 12 days, reaching several days (2–4) into the stationary phase. The culture was then exposed to atmospheric concentrations of CH_4_ for one additional day during which the number of cells in the culture was estimated using cell counts. Then, the cells were filtered out and the filters were prepared for extractions. With this approach, our aim was to extract DNA from cells in a slow growth state where the cells do not contain multiple partial genomes. Filters for the standard and those from the experiment incubation were cut into 12 pieces and put into 2 mL safelock tubes, after which DNA was extracted from the filters using DNA IQ™ Casework Pro Kit for Maxwell^®^ 16 following the manufacturer’s instructions. In brief, we crushed the 12 filter pieces with a metal bead in a tissue lyser (Qiagen, Hilden, Germany) six times, 30 s each, applying liquid nitrogen prior to the first and between each round in the tissue lyser. Prior to extraction, the crushed pieces were spun down. After, 400 µL of extraction buffer containing proteinase K and thioglycerol followed by 400 µL lysis buffer was added. Then, the mixture was transferred into a cartridge of the Maxwell 16 system and processed. DNA extracts were quantified using Qubit (dsDNA HS Assay, Thermo Fisher, Waltham, MA, USA). Cell numbers on filters from the experiment were subsequently determined by comparing the extract yields per filter to those of the standard, converting the values from nanograms of DNA to cell numbers.

### 2.5. Contamination Tests and Microscopy

In addition to the start of the pre-incubation and after eight months, we inspected filters by fluorescence microscopy at the beginning and end of the first gas uptake experiment. At the end of the experiment, one filter from each of the five treatments was used for contamination tests and fluorescence microscopy. For the second gas uptake experiment, we inspected filters at the beginning and end of the pre-incubation after 4.5 months, in addition to contamination tests after the experiment. Preparation for fluorescence microscopy was carried out as described for the pre-incubation above. Contamination tests were carried out by cutting filters in pieces and placing them on TGYA plates for 15 days. TGYA plates contained 5 g tryptone, 2.5 g of yeast extract, 1 g of glucose, and 20 g of agar per 1 L of water. Presence and growth of heterotrophic microorganisms was then evaluated by visual inspection. The contamination tests were negative and cell sizes and cell shapes were the same as prior to the experiment, confirming the purity of the culture.

### 2.6. Plotting and Statistics

All plotting was performed using the R [23] package ggplot.2 [24].

### 2.7. Cell-Specific Oxidation Rates and Free Energy Yield Calculations

Oxidation rates were estimated by first order rate kinetics models. We log transformed the concentrations over time and fitted linear regression models to the transformed plots. The slope of the linear models corresponds to the rate constants. By multiplying the respective rate constant by the atmospheric concentrations of CH_4_, H_2_ and CO, we obtained the rates of oxidation at atmospheric gas concentrations. The fit of the linear models was evaluated and considered to have satisfactory coefficients of determination, giving the following values: *R*^2^ − CH_4_ = 0.93, *R*^2^ − H_2_ = 0.82, *R*^2^ − CO = 0.68 (Experiment 1), and *R*^2^ − CH_4_ = 0.99, *R*^2^ − H_2_ = 0.99, *R*^2^ − CO = 0.99 (Experiment 2). The oxidation rates of H_2_ and CO were subsequently corrected by adjusting for the abiotic gas leakages. Due to the first order rate kinetics nature of the CO (*R*^2^ of linear models = 0.77, *n* = 5) and H_2_ (*R*^2^ of linear models = 0.99, *n* = 5) leakages, their respective rate constants were estimated the same way as for the oxidation rates. The rate constants for the biological H_2_ and CO oxidation were subsequently adjusted by subtracting or adding the rate constants of the leaks of H_2_ (leakage out of the bottle) and CO (leakage into), respectively. For each condition A, B, C, D and E, cell numbers on three filters were estimated. Cell-specific oxidation rates were subsequently calculated by dividing the estimated oxidation rates by the corresponding cell numbers.

The expected maintenance requirements at 20 °C were calculated according to Tijhuis et al. [8]. The Gibbs free energy changes (Δ_r_*G*) were calculated for the following reactions and Gibbs free energies of formation ΔG_f_° (kJ/mol), assuming atmospheric concentrations and 20 °C: [CH_4_ + 2O_2_ → CO_2_ + 2H_2_O]; [2CO + O_2_ → 2CO_2_]; [2H_2_ + O_2_ → 2H_2_O]. CO: −137.16 kJ/mol, O_2_: 0 kJ/mol, CO_2_: −394.39 kJ/mol, H_2_: 0 kJ/mol, H_2_O: −237.13 kJ/mol, CH_4_: −50.6 kJ/mol. From the oxidation rates and free energy change of the reactions, we could estimate the amount of energy obtained per mol of biomass carbon per hour, applying the dry weight and carbon content of *M. gorgona* MG08 (see below) as previously shown [2,6]. All calculations, raw data and literature data needed for input in the calculations are provided in a detailed format. The data and calculations are provided as excel formulas that can be intuitively followed and inspected. These can serve as a template for those wishing to repeat our calculations for similar experiments and compare those to our data (See Supplementary dataset Table S1, tabs “Experiment 1”, “Experiment 2”, “Gibbs_free_energy” and “Maintenance_energy”).

### 2.8. Estimating Cell Dry Weight

In order to estimate the dry weight of *M. gorgona* MG08, we cultivated 10 × 10 mL of *M. gorgona* MG08 culture as described for a young stationary culture in the cultivation section above. This provided ten replicates from which 1 mL was used for cell counts and 9 mL for drying overnight at 110 °C. Based on this, we calculated that the weight of one *M. gorgona* MG08 cell is 8.8 × 10^−14^ g dry weight per cell (SD = 3.1 × 10^−14^). We assume that half of the cell consists of carbon [2]. This weight and size (length 1.2 µm and width 0.7 µm) makes *M. gorgona* MG08 the lightest known Alphaproteobacteria MOB, compared to previously weighed strains [2]. While the size of *M. gorgona* MG08 cells do not change when growing with air as its energy and carbon source, compared to higher CH_4_ concentrations (it retains its size of length ~1.2 µm and width ~0.7 µm), we acknowledge that the content, density and thus dry weight of the cells growing in air may differ from that of *M. gorgona* MG08 cultivated at high CH_4_ concentrations. Such differences could affect the estimates of kJ Cmol h^−1^. However, due to the low biomass, we were not able to estimate cell weights from filters cultivated in air.

## 3. Results and Discussion

**Figure 1:**
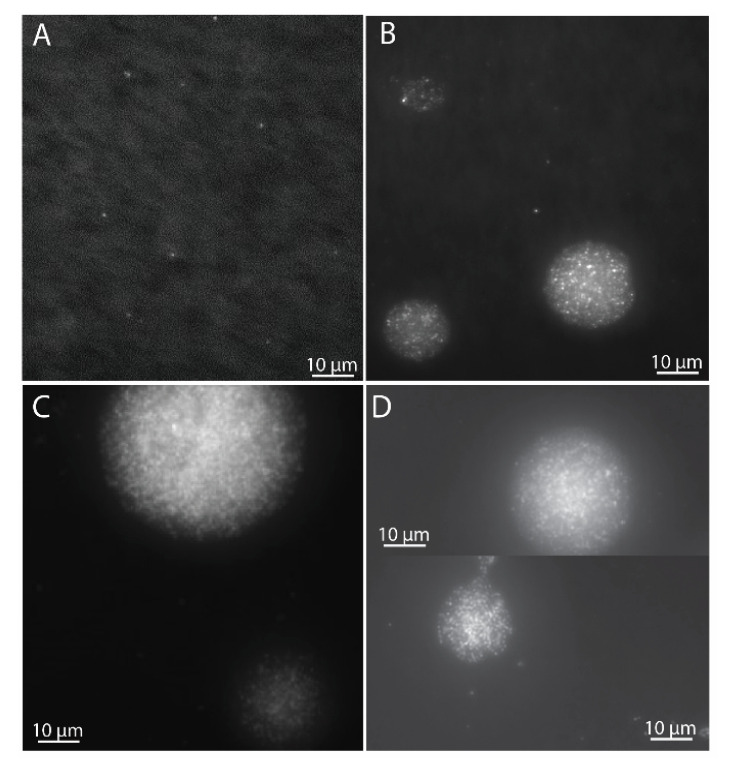
Microcolony growth of *M. gorgona* MG08 at different times during pre-incubation and gas uptake experiments. (**A**) Cells after filtration on polycarbonate filters, prior to incubation. (**B**) Microcolonies after 4 months of pre-incubation under ambient air. (**C**) Microcolonies after 8 months of pre-incubation under ambient air. (**D**) Microcolonies after 9 months of pre-incubation under ambient air and 57 days of gas uptake experiment incubation under synthetic air ((**D**), upper panel), and synthetic air + 2–4 p.p.m.v. CH_4_, CO and H_2_ ((**D**), lower panel). For fixation, the filters were transferred to fresh-made 2% paraformaldehyde in 1× PBS in the refrigerator overnight. For staining, filters were transferred (side with bacteria up) on top of 200 μL droplets of 1000× SYBRgreen I (10× dilution of the stock concentration provided by Thermo Fisher Scientific, Invitrogen, Molecular probes) and incubated for 10 min, washed, and air dried.

**Figure 2:**
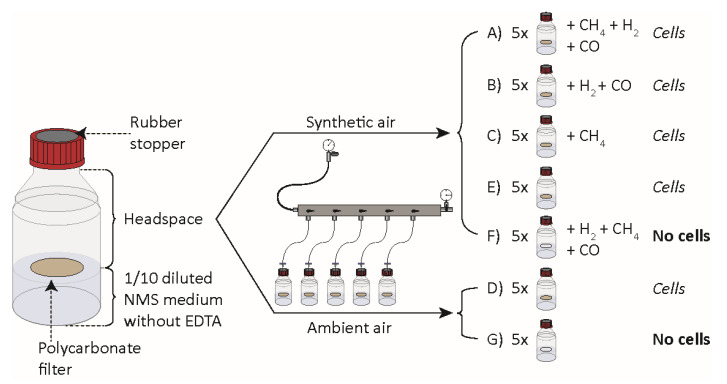
Experimental setup for experiment 1 to determine gas uptake by *M. gorgona* MG08. NMS: Nitrate mineral salts. Synthetic air containing less than 0.2 p.p.m.v. of CH_4_, CO and H_2_. Ambient air containing 1.84 p.p.m.v. CH_4_, 0.4 p.p.m.v. CO and 0.7 p.p.m.v. H_2_. All injections of CH_4_, CO and H_2_ resulted in a final concentration between 2 and 4 p.p.m.v of the respective gas.

Cells of *M. gorgona* MG08 were pre-incubated on filters under an atmosphere of ambient air (~1.84 p.p.m.v. CH_4_, ~0.39 p.p.m.v. CO and ~0.7 p.p.m.v. H_2_) for eight months. The filters floated on diluted mineral medium within bottles to enable optimal gas transfer. After four months, a subset of the cells had developed into microcolonies of more than 100 cells (Figure 1), while some cells had not divided. Visual inspection and counting showed that the number of generations in the majority of the colonies had surpassed seven (64 cells) (Figure 1C), matching previous observations [4]. Studies have shown that up to three generations can be supported by intracellular storages [4,25,26], confirming that the eight-month pre-incubation was sufficient to ensure growth with carbon and energy harvested solely from air. After nine months of pre-incubation, the filter-containing glass bottles were sealed with rubber stoppers before defined headspace atmospheres were created (Figure 2).

**Figure 3:**
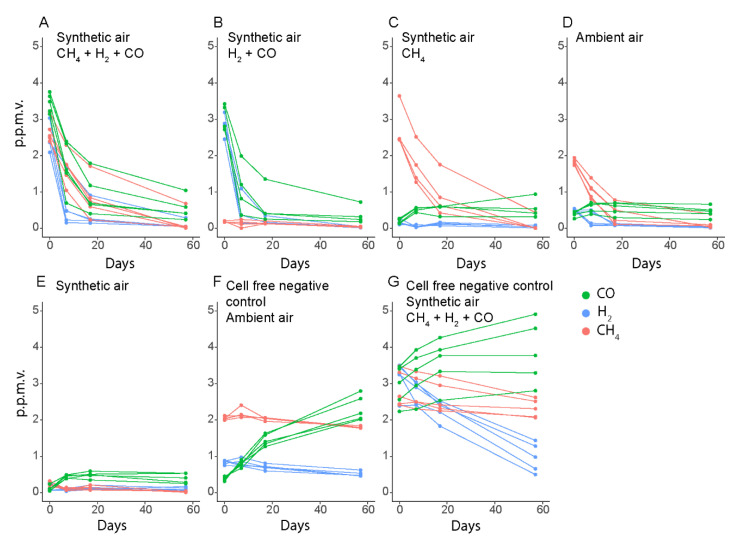
Oxidation of CH_4_, CO, and H_2_ by *M. gorgona* MG08. (**A**–**E**) contained polycarbonate filters with *M. gorgona* MG08 floating on nitrate mineral salts medium. (**F**,**G**) contained sterile filters. The lower H_2_ concentrations at the start of the ambient air incubations with cells (**D**) compared to those without cells (**F**) are due to leaving prepared bottles overnight before measuring the first time point T_0_, allowing some oxidation to already occur. All gases used were of the highest commercially available quality, 6.0 (99.9999% purity). The multiple data points in each color represent different biological replicates from the same condition. Synthetic air contained less than 0.2 p.p.m.v. of CH_4_, CO and H_2_.

In a 57-day experiment, we could show that *M. gorgona* MG08 is able to oxidize ambient air to sub-atmospheric concentrations of CH_4_ and H_2_ (Figure 3). In several of the incubations, CH_4_ and H_2_ concentrations reached below atmospheric levels already after 7 days, while CO concentrations did not decrease below ~0.45 p.p.m.v., which is close to the atmospheric levels in urban areas [16,17,18,19] and similar to the average ambient air concentrations measured in the laboratory (0.39 p.p.m.v.). Furthermore, the CH_4_ oxidation rates did not depend on the CO and H_2_ concentrations (Figure 3A,C,D), while CO and H_2_ oxidation proceeded without the presence of CH_4_ (Figure 3B).

**Figure 4:**
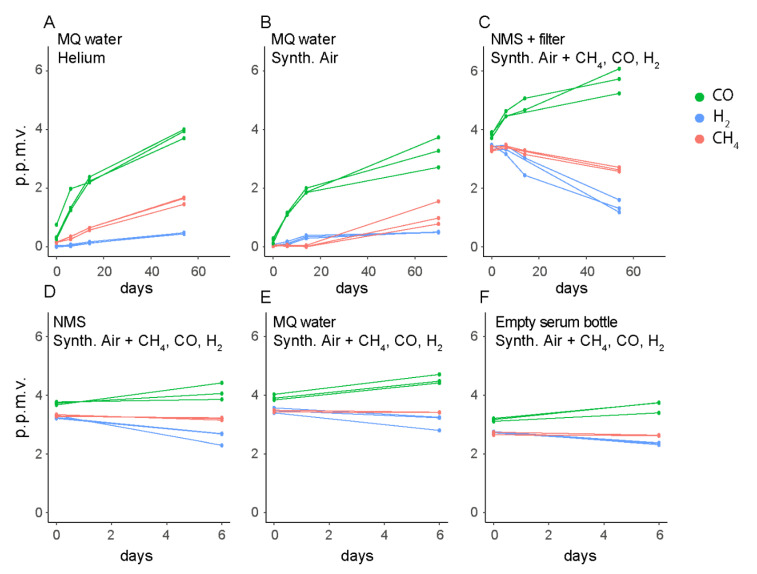
Gas leakage and other abiotic gas sources. (**A**,**B**) Long incubations of cell-free controls were performed to identify signs of CO and CH_4_ leakage or release into or out of the bottles. (**C**–**F**) Short incubations of cell-free controls were considered sufficient to identify the same patterns of gas leakage and accumulation irrespective of bottle liquid and headspace composition. MQ: MilliQ. Synth. Air: Synthetic air. NMS: nitrate mineral salts. The multiple data points in each color represent different biological replicates from the same condition. Synthetic air contained less than 0.2 p.p.m.v. of CH_4_, CO and H_2_.

The stabilization of the CO concentrations around 0.45 p.p.m.v. in bottles with *M. gorgona* MG08 cells, irrespective of the start concentrations, was unexpected (Figure 3A,B,D,E). In the cell-free controls, we observed increasing CO concentrations, reaching 2 p.p.m.v. during 57 days of incubation (Figure 3F,G), suggesting these observations to be due to an abiotic CO source. To identify the CO source, we measured CO, H_2_ and CH_4_ in several cell-free controls, including empty bottles (Figure 4). CO accumulation was detected in these liquid-free bottles, and even in headspaces exposed to only glass and rubber stoppers. Thus, we concluded that CO was released from the rubber stoppers, meaning that the stabilization of CO concentrations at ~0.45 p.p.m.v. in the presence of *M. gorgona* MG08 was a confirmation of continuous CO oxidation at near ambient air concentrations of ~0.45 p.p.m.v. The decreasing or increasing CH_4_ and H_2_ concentrations in abiotic controls (Figure 3F,G and Figure 4A–F), on the other hand, could be explained by leakage during long incubation times, as previously observed [27,28]. In addition to the fact that such long-term leakages have been observed previously, the rationale for this conclusion is that the concentrations inside the bottles always increased when the outside concentrations were higher, and decreased when the outside concentrations were lower.

### Cell-Specific Oxidation Rates and Energy Yield

In order to estimate the CH_4_, CO and H_2_ uptake rates by *M. gorgona* MG08 at atmospheric concentrations, we tested and confirmed that the rate change of CH_4_ uptake fulfilled the assumptions of first order rate kinetics (linear regression through the natural logarithm (LN) of rates over time *R*^2^ = 0.93, *n* = 15). As microcolonies contained more than one layer of cells, filter cell counts were not possible. Instead, we performed DNA extractions and cell quantification with a DNA to cell count standard for a subset of the incubation bottles, showing that all but one floating filter contained between 38.1 and 68 million cells (Table S1). Based on these estimates, we calculated the cellular CH_4_ oxidation rates at atmospheric CH_4_ concentrations to 0.7–2.8 × 10^−18^ mol cell^−1^ h^−1^ at 20 °C (*n* = 9). Similarly, the H_2_ and CO oxidation rates at atmospheric concentrations (average H_2_ linear model *R*^2^ = 0.82, *n* = 10, CO R^2^ = 0.68, *n* = 10) were estimated to be 0.17–0.36 and 0.20–0.34 × 10^−18^ mol cell^−1^ h^−1^, respectively (*n* = 6). These rates were corrected for the leakage and release of H_2_ and CO, respectively. The CH_4_ oxidation rates reflect the lower end of the atmospheric oxidation rates estimated for conventional MOB, which range from 0.2 to 17 × 10^−18^ mol cell^−1^ h^−1^ at 25 °C [6]. However, the rates of conventional MOB forming the basis for these estimates were measured at high CH_4_ concentrations and thus do not necessarily represent the rates that would be obtained at atmospheric conditions.

Based on the specific affinity estimated from oxidation rates at higher than atmospheric concentrations, the expected cell-specific CH_4_ oxidation rate at atmospheric concentrations can be calculated [2]. From the oxidation rates measured at concentrations between 823 p.p.m.v. and 6% [4], we found that *M. gorgona* MG08 would have a cell specific CH_4_ oxidation rate of 10 × 10^−18^ mol cell^−1^ h^−1^ when growing at atmospheric concentrations. However, these estimates are more than five times higher than the actual rate measurements at atmospheric concentrations provided in our current study. Thus, *M. gorgona* MG08 may not sustain the same catalytic properties or amount of particulate methane monooxygenase (pMMO) enzymes per cell during growth in air. At concentrations between 823 p.p.m.v. and 6%, we estimated the K_m(app)_ of *M. gorgona* MG08 to be 4.905 [4], similar to that of various, presumed low affinity, MOB [2]. Although this indicated that *M. gorgona* MG08 has a low affinity for CH_4_, the dependency of K_m(app)_ on the V_max_ prevents us from determining the exact affinity unless we perform comparative kinetic experiments for purified enzymes from several MOB strains, or compare the CH_4_ oxidation rates and pMMO concentrations of several different MOB. Thus, it seems plausible that life at atmospheric CH_4_ concentrations can be sustained by low affinity enzymes, but it is still uncertain whether this is the case.

Based on the specific affinity estimated from oxidation rates at higher than atmospheric concentrations, the expected cell-specific CH_4_ oxidation rate at atmospheric concentrations can be calculated [2]. From the oxidation rates measured at concentrations between 823 p.p.m.v. and 6% [4], we found that *M. gorgona* MG08 would have a cell specific CH_4_ oxidation rate of 10 × 10^−18^ mol cell^−1^ hour^−1^ when growing at atmospheric concentrations. However, these estimates are more than five times higher than the actual rate measurements at atmospheric concentrations provided in our current study. Thus, *M. gorgona* MG08 may not sustain the same catalytic properties or amount of particulate methane monooxygenase (pMMO) enzymes per cell during growth in air. At concentrations between 823 p.p.m.v. and 6%, we estimated the K_m(app)_ of *M. gorgona* MG08 to be 4.905 [4], similar to that of various, presumed low affinity, MOB [2]. Although this indicated that *M. gorgona* MG08 has a low affinity for CH_4_, the dependency of K_m(app)_ on the V_max_ prevents us from determining the exact affinity unless we perform comparative kinetic experiments for purified enzymes from several MOB strains, or compare the CH_4_ oxidation rates and pMMO concentrations of several different MOB. Thus, it seems plausible that life at atmospheric CH_4_ concentrations can be sustained by low affinity enzymes, but it is still uncertain whether this is the case.

The energy yields per mol of CH_4_, CO and H_2_ under the provided experimental conditions (ambient air at 20 °C) were approximately −814, −522 and −472 kJ/mol, respectively, assuming the following reactions: [CH_4_ + 2O_2_ → 2H_2_O + CO_2_], [2CO + O_2_ → 2CO_2_], [2H_2_ + O_2_ → 2H_2_O]. Based on the measured rates, we find that *M. gorgona* MG08 is able to conserve approximately 0.47 kJ Cmol h^−1^ from the combined oxidation of CH_4_, H_2_ and CO during growth in air at 20 °C (see data and full calculation in Table S1). This calculation implements our dry weight estimates for *M. gorgona* MG08 (Table S1). Interestingly, these estimates show that *M. gorgona* MG08 cells (length ~1.2 µm and width ~0.7 µm) are three times lighter (0.88 × 10^−13^ gDW cell^−1^) than the lightest reported MOB [2]. The mass of a cell could affect its energy budget, as more energy might be needed to supply maintenance in a heavier cell, leaving less for growth. Additionally, a heavier cell might require more energy for growth. Thus, the low weight of *M. gorgona* MG08 may reflect a strategy to reduce energy costs for growth. In contrast to our findings, the previously reported soil CH_4_ oxidation rates of 800 × 10^−18^ mol cell^−1^ h^−1^ [6] would provide 179 kJ Cmol h^−1^. In order to examine this contradiction and test the validity of our data, we repeated parts of our experiment (condition with 5 p.p.m.v. of each gas CH_4_, H_2_ and CO) with shorter pre-incubation (4.5 months) to minimize growth stagnation and the possibility for the accumulation of dead cells on the floating filters.

**Figure 5:**
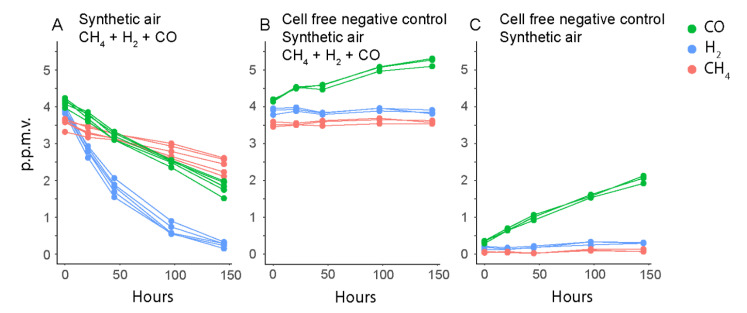
Oxidation of CH_4_, CO, and H_2_ by *M. gorgona* MG08. (**A**) filters with *M. gorgona* MG08 floating on nitrate mineral salts medium. (**B**,**C**) sterile filters floating on nitrate mineral salts medium. All gases used were of the highest commercially available quality, 6.0 (99.9999% purity). The multiple data points in each color represent different biological replicates from the same condition.

Furthermore, we shortened the gas uptake experiment to less than one week of incubation to prevent gas leakage effects on rate estimates. This new setup provided highly precise estimates of CH_4_, H_2_ and CO uptake rates (Figure 5), which confirmed their first order nature (linear regression through LN of rates over time; CH_4_ *R*^2^ = 0.99, *n* = 5; H_2_ *R*^2^ = 0.99; CO *R*^2^ = 0.99). Within this timeframe, the leakage of H_2_ and CH_4_ was negligible (Figure 5B,C), but the release of CO from the rubber stoppers occurred and was corrected for. Interestingly, similar but slightly lower cellular oxidation rates at atmospheric gas concentrations were estimated. The CH_4_ uptake ranged from 0.32 to 1.3 × 10^−18^ mol cell^−1^ h^−1^, the H_2_ uptake from 0.63 to 2.1 × 10^−18^ mol cell^−1^ h^−1^, and the CO uptake from 0.24 to 0.69 × 10^−18^ mol cell^−1^ h^−1^ at 20 °C. We suspect the differences were due to the higher accuracy of the uptake rates, and higher cell number estimates (average 9.54 × 10^7^; Table S1), despite shorter incubation time (4.5 months). The number of initial cells that form colonies on the filters may vary between experiments, possibly due to small differences in the physiological state of the inoculum. This, and the possibility that colonies of a certain size reach a growth stagnation phase due to limiting concentrations of CH_4_, H_2_ and CO around the colony, can explain how additional pre-incubation time in the first experiment did not lead to higher cell numbers per filter than in the second experiment.

With these new numbers, we find that *M. gorgona* MG08 is able to conserve approximately 0.38 kJ Cmol h^−1^ (close to the 0.48 kJ Cmol h^−1^ estimated from the first experiment) during growth in air at 20 °C (Table S1). This is more than seven times lower than the estimated average maintenance requirement of a bacterial population at 20 °C (2.8 kJ Cmol h^−1^) [8]. However, this amount of energy is apparently sufficient to sustain the growth of *M. gorgona* MG08. Thus, we question whether the average maintenance requirement of bacteria [8] appropriately describes the constraints for growth on atmospheric trace gases by *M. gorgona* MG08 and other atmMOB.

With our numbers, the 2.8 kJ Cmol h^−1^ minimum requirement mentioned above could be achieved if approximately 14 million out of the ~100 million cells on a filter were active [8]. Alternatively, if cell density and thus weight (cell sizes do not seem to vary with CH_4_ concentration) were lower during growth in air, an energy yield of 2.8 kJ Cmol h^−1^ or higher, could be achieved. The reason is that the same amount of energy would be distributed on less cellular mass than assumed for our empirical estimate of 0.38 kJ Cmol h^−1^. Regardless, the rates of *M. gorgona* MG08 are substantially lower than those in high upland soils, where CH_4_ oxidation rates of up of 800 × 10^−18^ mol cell^−1^ h^−1^, almost 500 times faster than *M. gorgona* MG08, were measured. However, these were based on soil cell numbers estimated from DNA extractions and *pmoA* qPCR [6]. Thus, the numbers have possibly been underestimated, as DNA extractions from soils may provide less than 100% yields, qPCR quantification can be inhibited by soil-derived impurities in the DNA extract, and primer mismatches can result in an underrepresentation of copy numbers. Methanogenic archaea and acetogenic bacteria were recently found to require much less maintenance energy (0.2 kJ Cmol h^−1^) than previously believed (9.8 kJ Cmol h^−1^) [29]. According to the authors, the low maintenance energy was based on the low growth rates of these organisms, a feature that had not previously been taken into account. If true, low maintenance requirements at low growth rates could also explain how *M. gorgona* MG08 can sustain its slow growth at a limited energy budget.

We conclude that *M. gorgona* MG08 oxidizes the atmospheric trace gases CH_4_, CO and H_2_ to harvest energy for growth in air. The ability of *M. gorgona* MG08 to grow using air as its only source of energy and carbon relies not only on this metabolic flexibility, but also on its low energy requirements. Our findings suggest that a high CH_4_ affinity is not a prerequisite to live on atmospheric CH_4_.

## Supplementary Materials

The following are available online at https://www.mdpi.com/2076-2607/9/1/153/s1, Table S1: Supplementary data and calculations.
